# Incidence of Organic Acid Disorders in 13 Million Chinese Newborns: A Systematic Review and Meta-Analysis

**DOI:** 10.3390/ijns11040113

**Published:** 2025-12-13

**Authors:** Shuting Huang, Qiongfang Yao, Fei Kong, Min Wu, Xiaolong Qiu, Peiran Zhao, Yinglin Zeng, Jinying Luo, Liangpu Xu, Jinfu Zhou

**Affiliations:** 1 Fujian Maternity and Child Hospital College of Clinical Medicine for Obstetrics & Gynecology and Pediatrics, Fujian Medical University, Fuzhou 350001, China; m18350320353@163.com (S.H.); yqfyqf0703@163.com (Q.Y.); kf20000723@163.com (F.K.); 18706385091@163.com (M.W.); 15394536832@163.com (X.Q.); zhaopeiran3@163.com (P.Z.); jiandanchn@163.com (Y.Z.); 2 Department of Preventive Medicine, School of Public Health, Fujian Medical University, Fuzhou 350122, China; 3 School of Medical Technology and Engineering, Fujian Medical University, Fuzhou 350122, China

**Keywords:** incidence, meta-analysis, neonatal screening, organic acid disorders, China

## Abstract

Organic acid disorders (OADs) are inherited metabolic defects in the enzymes and cofactors involved in metabolic pathways. This systematic review and meta-analysis investigated the incidence and regional differences in OADs between the northern and southern regions of China. Searches of the PubMed, Embase, Web of Science, and Chinese databases (CNKI, Veipu, and Wanfang) revealed 1784 studies indexed between January 2002 and December 2024. After quality assessment and data extraction, the meta-analysis was conducted on OAD screening data from 57 studies involving 13,314,056 newborns and 1501 OAD cases in China. The seven most prevalent OADs were methylmalonic acidemia (MMA), 3-methylcrotonyl-CoA carboxylase deficiency, glutaric acidemia type I, isobutyryl-CoA dehydrogenase deficiency, isovaleric acidemia, 2-methylbutyryl-CoA dehydrogenase deficiency (2-MBD), and propionic acidemia. The meta-analysis revealed an OAD prevalence of 112.38 (95% confidence interval 106.70–118.07) per 1,000,000 newborns. The incidence of OADs and MMA was significantly higher in northern China than in southern China, whereas the incidence of 2-MBD was significantly lower in northern China than in southern China (*p* < 0.0001). Additionally, the ratio of MMA combined with homocystinuria to MMA was higher in northern China than in southern China (*p* < 0.05). These results provide valuable epidemiological insights and guidance for newborn screening for OADs in China.

## 1. Introduction

Organic acid disorders (OADs) are a group of genetic disorders that result from defects in the enzymes and cofactors involved in metabolic pathways. Due to the accumulation of toxic substrates or intermediate metabolites and insufficiency of terminal products, patients with OAD can present with clinical manifestations such as irreversible intellectual impairment, physical disabilities, and even death [1]. However, the clinical manifestations in affected babies are usually asymptomatic at birth or complex and often non-specific. Thus, early diagnosis and timely intervention for many OADs are crucial for preventing adverse complications in affected individuals.

Newborn screening (NBS) for inborn errors of metabolism (IEMs) is an essential public health program that enables early diagnosis, and it is both effective and cost-efficient. Tandem mass spectrometry (MS/MS) offers high sensitivity and specificity, along with a low sample volume (a single blood spot); thus, this tool has been widely employed in NBS for the detection of IEMs, including OADs, amino acid disorders, fatty acid oxidation disorders, and urea circulatory disorders.

In mainland China, MS/MS-based NBS was first implemented in 2002, with a pilot study reporting an average incidence of OADs of 1 in 8071 newborns [2]. However, subsequent studies have shown that the incidence and disease spectrum of OADs vary significantly across regions in China, particularly between the southern and northern regions, with rates ranging from 1:50,000 to 1:2300 in newborns [3,4,5]. Determining the overall and varying incidence of OADs in the Chinese population is crucial for guiding NBS policies and healthcare planning, particularly considering the potential regional disparities across the Qinling Mountains–Huaihe River Line. This north–south divide is associated with differences in genetic background, environment, geography, and lifestyle, all of which may influence disease incidence.

Therefore, the present meta-analysis comprehensively investigated the epidemiological characteristics of OADs in Chinese populations, analyzed the nationwide incidence rate of OADs, and elucidated the differences in the incidence and disease spectrum of OADs between the northern and southern regions.

## 2. Materials and Methods

### 2.1. Literature Search

Systematic reviews and meta-analyses were conducted following the Preferred Reporting Items for Systematic Reviews and Meta-Analyses guidelines [6], and the protocol was registered with PROspective Systematic Review PROtocols (ID: CRD420251132929; https://www.crd.york.ac.uk/PROSPERO/view/CRD420251132929, accessed on 31 August 2025). Three independent researchers (J.Z., J.L., and Y.Z.) systematically searched observational research databases on NBS for OADs between January 2002 and December 2024. The search included English databases such as PubMed, Embase, and Web of Science, as well as Chinese databases, including the China National Knowledge Infrastructure, Veipu, and Wanfang. The search terms were (“organic acid disorders” OR “organic acid metabolic disorders” OR “inborn errors of metabolism”) AND (“newborn screening”) AND (“China” OR “Chinese”).

### 2.2. Eligibility and Exclusion Criteria

Studies were included in the current review if they met the following criteria: (1) original observational studies; (2) studies reporting results of NBS for OADs in different cities, provinces, and autonomous regions of China; (3) study periods between 2002 and 2024; and (4) studies of relatively high quality.

Studies that did not meet these criteria were excluded. Additionally, (1) duplicate publications; (2) studies with overlapping screening regions or times (the study with more participants was included); (3) studies reporting only solo diseases of OADs; (4) studies of hospitalized newborns; and (5) studies not published in English or Chinese were also excluded.

All cases of organic acidemias showed abnormal results via MS/MS-based NBS and were further confirmed by genetic testing.

### 2.3. Data Extraction

Two researchers (S.H. and Q.Y.) independently extracted the data into an extraction table. The information obtained from the original publications included the first author, publication year, time period, geographic region, number of NBS participants, disease spectrum of the OADs, and the number of cases diagnosed with OADs. The estimated incidence rates of OADs were calculated using these extracted data. Discrepancies were resolved through discussions with another investigator (J.Z.). Since all data were based on previously published studies, ethical approval or patient consent was not required.

### 2.4. Quality Assessment

The quality of observational studies included in this meta-analysis was independently evaluated by two investigators (S.H. and Q.Y.) using the Agency for Healthcare Research Quality (AHRQ) criteria (Table S1). Studies meeting each criterion were scored as 1, whereas those not meeting or having uncertain criteria were scored as 0, resulting in a total score ranging from 0 to 11. Higher scores denoted superior quality, with studies with scores of 8–11, 4–7, and 0–3 points classified as high, moderate, or low quality, respectively. Any inconsistencies were discussed comprehensively and resolved by another reviewer (J.Z.).

### 2.5. Statistical Analyses

We performed a meta-analysis to estimate the pooled incidence and 95% confidence interval (CI) of OADs in China. All statistical analyses were performed using RevMan version 5.3 (Update Software Ltd., Oxford, UK). The chi-square test and *I*^2^ statistic were used to evaluate the statistical heterogeneity among the studies. For comparisons with *I*^2^ values < 50% and *p*-values > 0.10, a fixed-effects model using the Mantel–Haenszel method was used to calculate the pooled incidence along with the odds ratio (OR) and 95% CIs. Otherwise, a random-effects model using the Der Simonian and Laird method was used.

We also performed a subgroup analysis to assess the effects of geographic region across studies. Significant differences in subgroup comparisons and disparities were defined as those with *p* < 0.05.

Sensitivity analyses were conducted by systematically excluding individual studies to assess their impact on the pooled ORs. Publication bias was visually assessed using a funnel plot and quantitatively evaluated using Begg’s test, with *p* < 0.1 indicating publication bias.

## 3. Results

### 3.1. Study Selection

**Figure 1:**
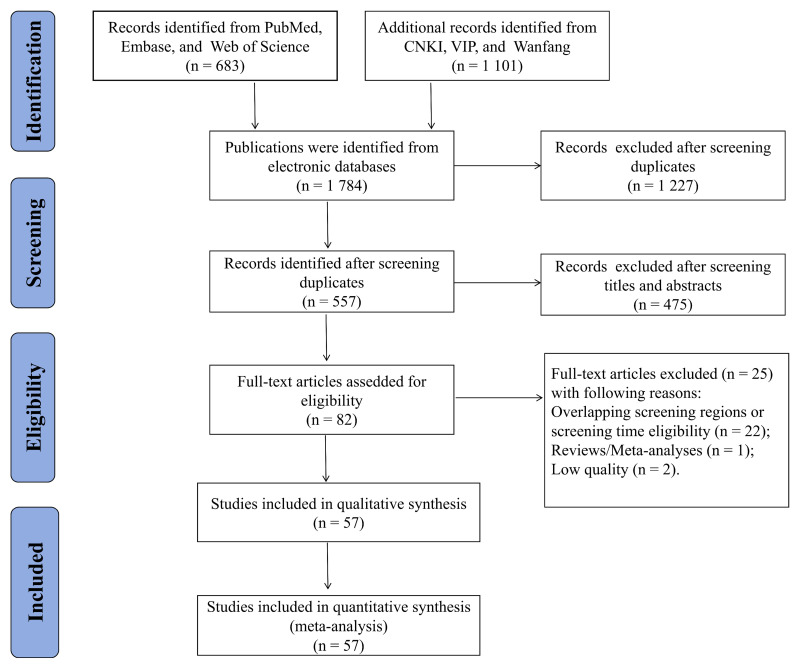
Flow chart of the study selection process.

The initial database searches identified 1784 articles from the six databases, including 683 in Chinese and 1101 in English. After screening for duplicates, 1227 articles were excluded, and 557 articles remained. An additional 475 articles were excluded because they did not meet the inclusion criteria after a review of their titles and abstracts. Subsequently, 82 articles were considered potentially eligible and underwent a thorough full-text review. Overall, 25 articles were excluded for overlapping screening or screening time eligibility regions (22 articles), study design (one meta-analysis), and low quality (two articles). Finally, the meta-analysis included 57 eligible studies. A flowchart of the literature search and processing is shown in Figure 1.

### 3.2. Study Characteristics

**Figure 2:**
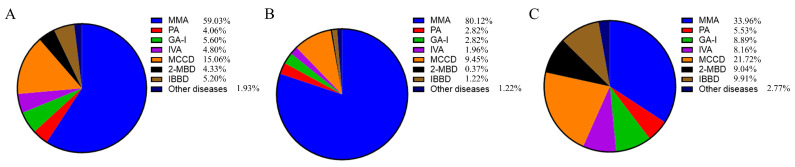
Disease spectrum of OADs in Chinese newborns. (**A**) All regions. (**B**) Northern region. (**C**) Southern region. OAD, organic acid disorder.

The 57 included studies involved 13,314,056 newborns, 1501 of whom were diagnosed with OADs (Table 1). Notably, 67.11% (8,935,954/13,314,056) of screened newborns resided in southern China. Among the 1501 patients with OADs, the seven most prevalent diseases accounted for 98.07% of the total number; these OADs included methylmalonic acidemia (MMA; 59.03%, 886/1501), 3-methylcrotonyl-CoA carboxylase deficiency (MCCD; 15.06%, 226/1501), glutaric acidemia type I (GA-I; 5.60%, 84/1501), isobutyryl-CoA dehydrogenase deficiency (IBDD; 5.20%, 78/1501), isovaleric acidemia (IVA; 4.80%, 72/1501), 2-methylbutyryl-CoA dehydrogenase deficiency (2-MBD; 4.33%, 65/1501), and propionic acidemia (PA; 4.06%, 61/1501; Figure 2A). The remaining 29 cases of OADs included 11 cases of holocarboxylase synthetase deficiency, three cases of biotinidase deficiency, three cases of 3-methylglutaconic aciduria type I, nine cases of 3-hydroxy-3-methylglutaryl-CoA lyase deficiency, two cases of ethyl malonic encephalopathy, and one case of malonic acidemia. MMA accounted for up to 80.12% (653/815) of all OADs in northern China, whereas the proportions of MMA and MCCD in northern China were 33.96% (223/686) and 21.72% (149/686), respectively (Figure 2B,C). Of the 669 cases of MMA for which subtype data were available, 95 were isolated MMA and 574 were MMA combined with homocystinuria.

### 3.3. The Result of Quality Assessment

According to AHRQ assessment items, articles with scores ≥ 4 were classified as moderate or high quality. The average score was 7.73, indicating minimal risk of bias (Table 1).

### 3.4. Meta-Analysis Results

#### 3.4.1. OAD Incidence

**Figure 3:**
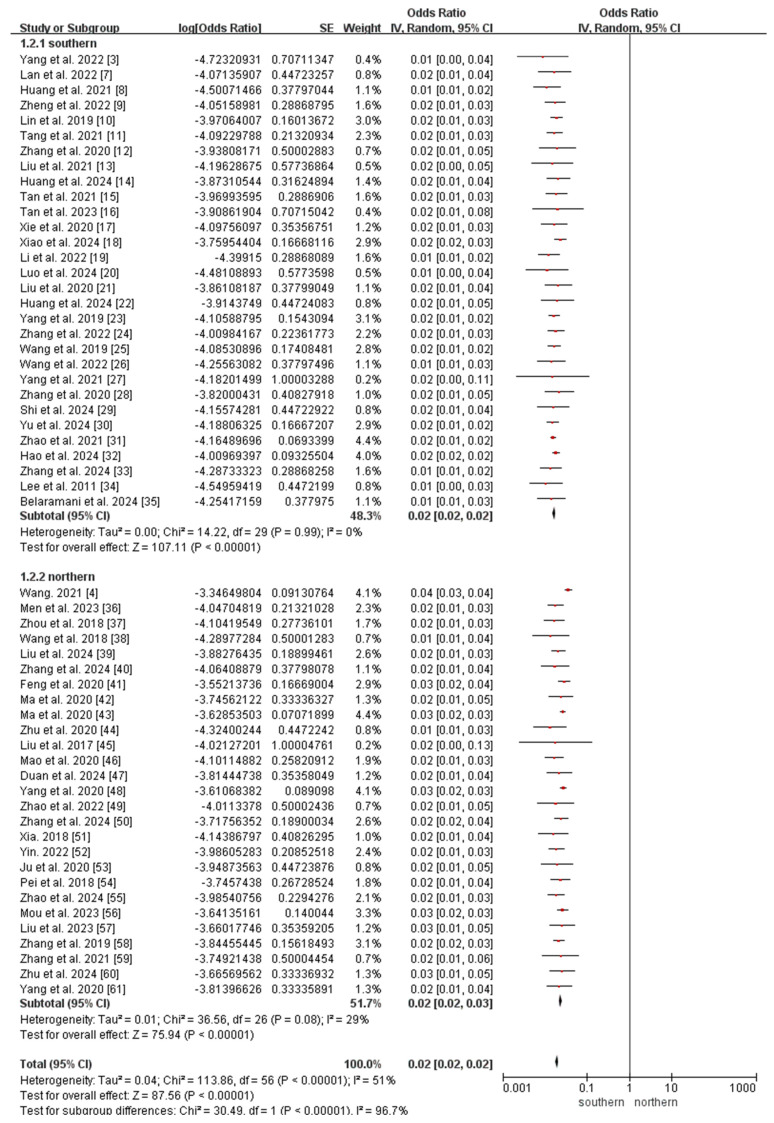
Meta-analysis of the prevalence of OADs between southern and northern China. OAD, organic acid disorder [3,4,7,8,9,10,11,12,13,14,15,16,17,18,19,20,21,22,23,24,25,26,27,28,29,30,31,32,33,34,35,36,37,38,39,40,41,42,43,44,45,46,47,48,49,50,51,52,53,54,55,56,57,58,59,60,61].

All included studies reported the incidence of OADs. Owing to the significant heterogeneity among the included studies (*I*^2^ = 51%, *p* < 0.05), a random-effects model was used to analyze the incidence of OADs in China. The meta-analysis revealed that the incidence of OADs was 112.38 (95% CI 106.70–118.07) per 1,000,000 newborns in China (Figure 3).

**Figure 4:**
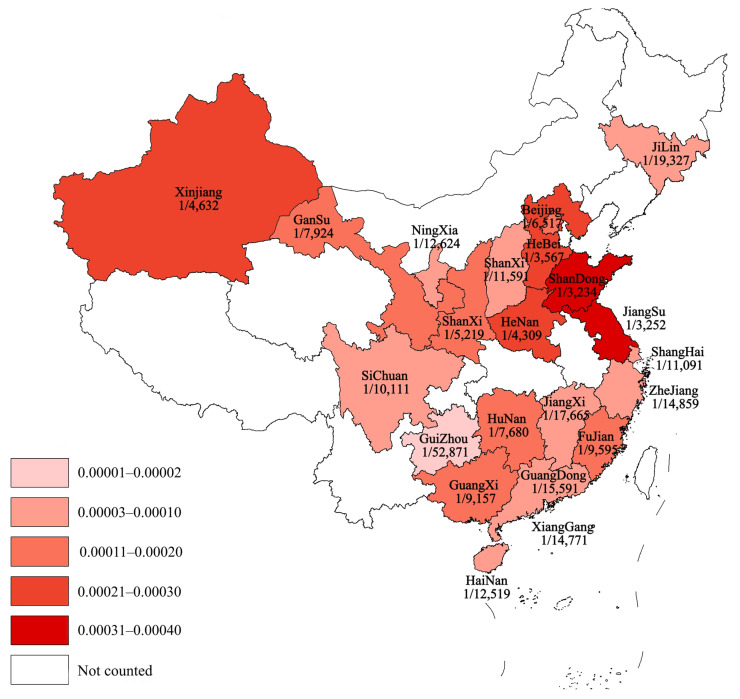
Schematic diagram showing the prevalence of OADs in different provinces of China. OAD, organic acid disorder.

Subgroup analyses of regional incidence of OADs revealed a significantly higher incidence in northern China (184.40 per 1,000,000, 95% CI 171.74–197.06) than in southern China (76.77 per 1,000,000, 95% CI 71.02–82.51; *p* < 0.0001; Figure 3 and Figure 4).

#### 3.4.2. Incidence of OAD Disease Spectrum

We also performed a meta-analysis of the seven most prevalent OADs, including MMA, MCCD, GA-I, IBDD, IVA, 2-MBD, and PA. As significant heterogeneity was observed among the included studies (*I*^2^ = 63%, *p* < 0.001), we used a random-effects model to analyze the incidence of MMA. No significant heterogeneity was identified in the incidence of the other six diseases (*I*^2^ = 0%, *p* > 0.05); therefore, we applied a fixed-effects model for further analysis. The meta-analysis results showed incidences of MMA, MCCD, GA-I, IBDD, IVA, 2-MBD, and PA of 66.34 (95% CI 61.97–70.71), 16.92 (95% CI 14.72–19.13), 6.29 (95% CI 4.94–7.93), 5.84 (95% CI 4.54-7.14), 5.39 (95% CI 4.15–6.64), 4.87 (95% CI 3.68–6.05), and 4.57 (95% CI 3.42–5.71) per 1,000,000, respectively.

**Figure 5:**
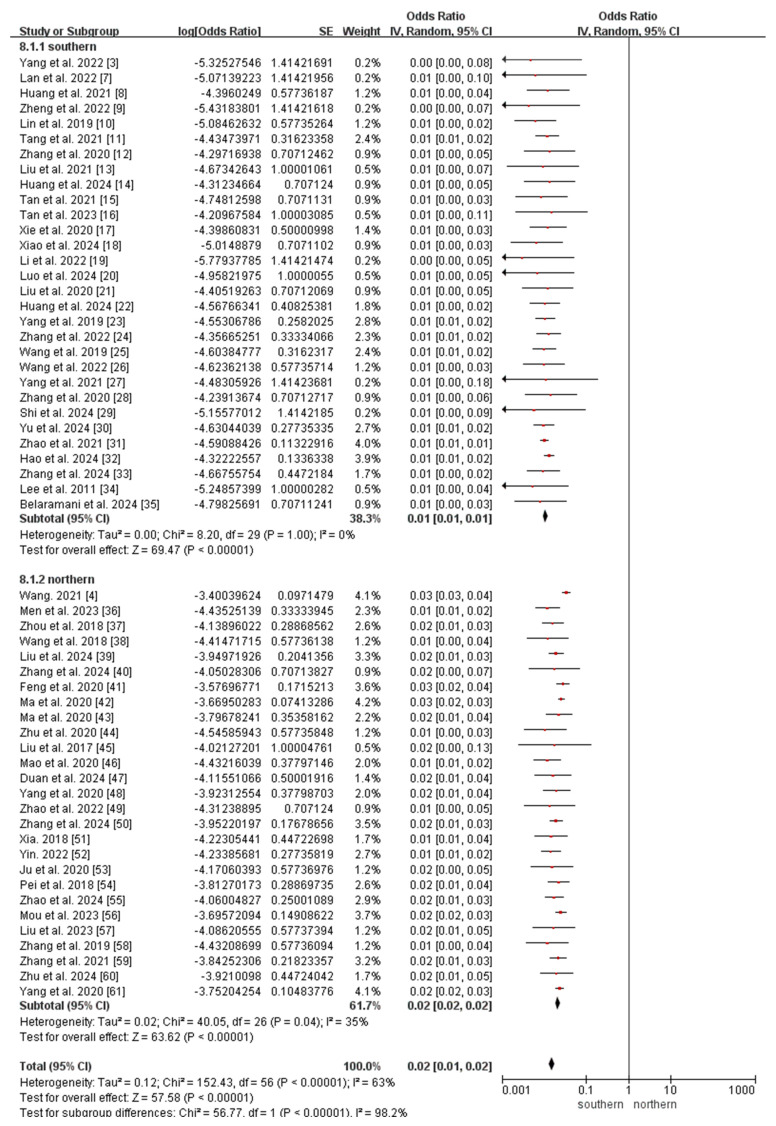
Meta-analysis of the prevalence of MMA between southern and northern China. MMA, methylmalonic acidemia [3,4,7,8,9,10,11,12,13,14,15,16,17,18,19,20,21,22,23,24,25,26,27,28,29,30,31,32,33,34,35,36,37,38,39,40,41,42,43,44,45,46,47,48,49,50,51,52,53,54,55,56,57,58,59,60,61].

**Figure 6:**
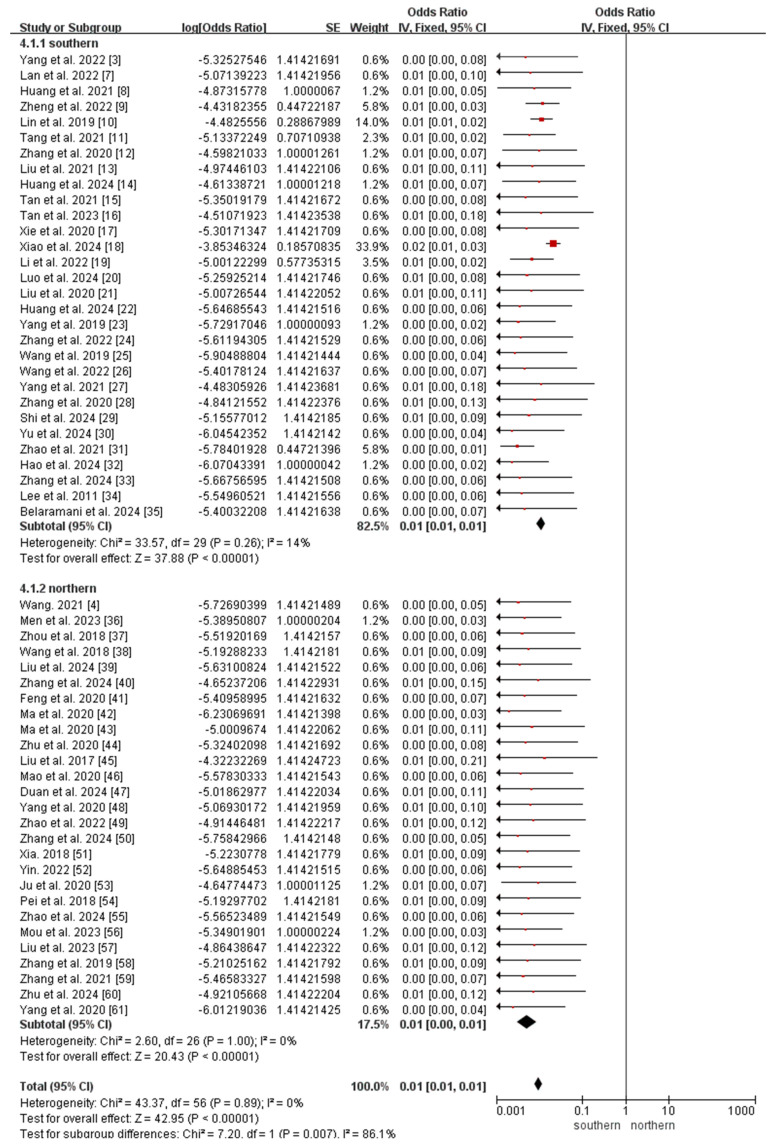
Meta-analysis of the prevalence of 2-MBD between southern and northern China. 2-MBD, 2-methylbutyryl-CoA dehydrogenase deficiency [3,4,7,8,9,10,11,12,13,14,15,16,17,18,19,20,21,22,23,24,25,26,27,28,29,30,31,32,33,34,35,36,37,38,39,40,41,42,43,44,45,46,47,48,49,50,51,52,53,54,55,56,57,58,59,60,61].

The subgroup analyses revealed that the incidence of MMA in northern China (147.74 per 1,000,000, 95% CI 136.41–159.08) was significantly higher than that in southern China (26.07 per 1,000,000, 95% CI 22.73–29.42; *p* < 0.0001; Figure 5), whereas the incidence of 2-MBD in northern China (0.68 per 1,000,000, 95% CI 0.09–1.45) was significantly lower than that in southern China (6.94 per 1,000,000, 95% CI 5.21–8.67; *p* < 0.0001; Figure 6). The incidence of the other diseases did not differ significantly between southern and northern China (Figures S1–S5).

**Figure 7:**
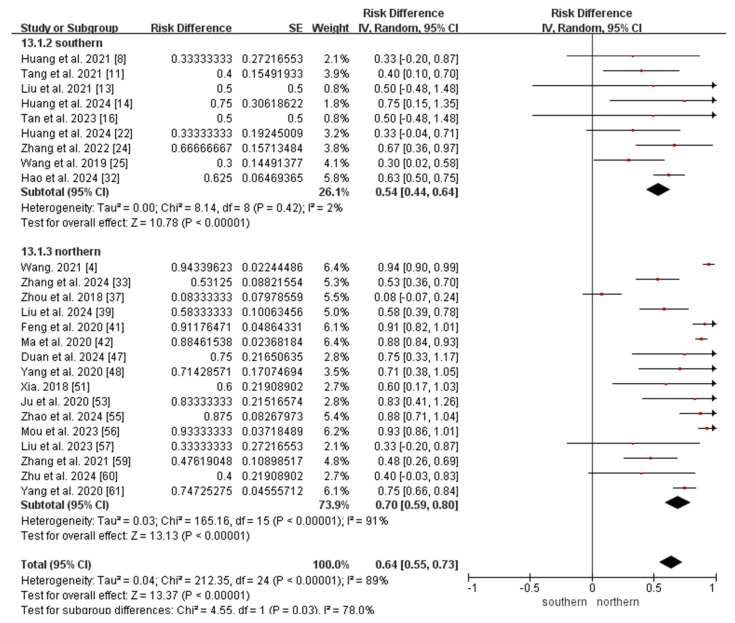
Meta-analysis of the ratio of MMA combined with homocystinuria to MMA between southern and northern China. MMA, methylmalonic acidemia [4,8,11,13,14,16,22,24,25,32,33,37,39,41,42,47,48,51,53,55,56,57,59,60,61].

Finally, the meta-analysis of the ratio of MMA combined with homocystinuria to MMA included 25 studies (9 from southern China and 16 from northern China). Owing to the substantial heterogeneity observed among the included studies (*I*^2^ = 89%, *p* < 0.05), a random-effects model was applied. The result showed that the ratio of MMA combined with homocystinuria to MMA was 0.64 (95% CI 0.55–0.73). The results of the subgroup analyses revealed that the ratio of MMA combined with homocystinuria to MMA in northern China (0.70, 95% CI 0.59–0.80) was higher than that in southern China (0.54, 95% CI 0.44–0.64; *p* < 0.05; Figure 7).

#### 3.4.3. Publication Bias

**Figure 8:**
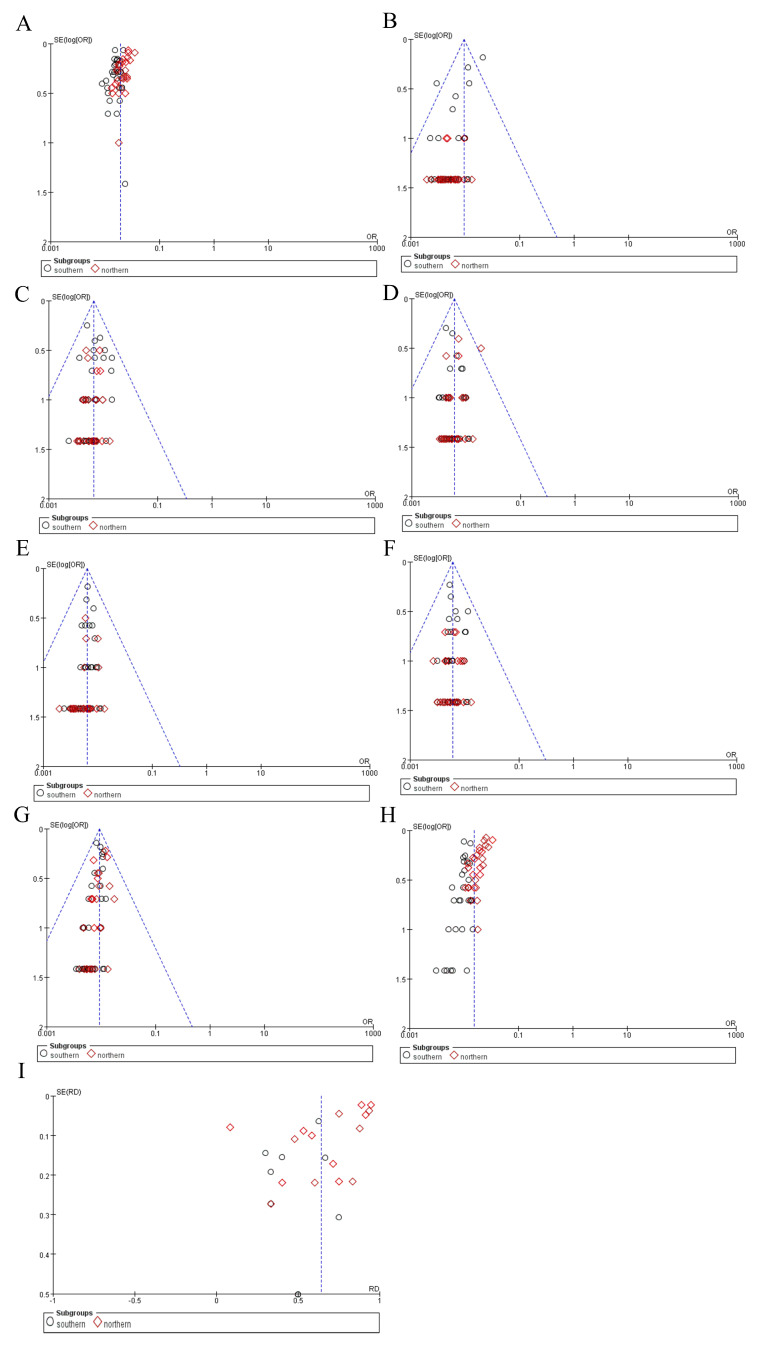
Funnel plots for publication bias. (**A**) OAD incidence. (**B**) 2-MBD incidence. (**C**) GA-I incidence. (**D**) PA incidence. (**E**) IBBD incidence. (**F**) IVA incidence. (**G**) MCCD incidence. (**H**) MMA incidence. (**I**) Ratio of MMA combined with homocystinuria to MMA. OAD, organic acid disorder; 2-MBD, 2-methylbutyryl-CoA dehydrogenase deficiency; GA-I, glutaric acidemia type I; PA, propionic acidemia; IBBD, isobutyryl-CoA dehydrogenase deficiency; IVA, isovaleric acidemia; MCCD, 3-methylcrotonyl-CoA carboxylase deficiency; MMA, methylmalonic acidemia.

A funnel plot, a scatterplot commonly used in meta-analyses, was generated to visually assess the presence of publication bias and other small-study effects. The funnel plots regarding the incidence of OADs were generally symmetrical, indicating no significant publication bias (Figure 8).

## 4. Discussion

This meta-analysis included 57 studies spanning the past 20 years, covering over 13 million newborns screened for neonatal OADs across 22 provinces (municipalities) in China. To date, this is the most comprehensive systematic review of OAD screening in China. Our study revealed an OAD incidence of 112.38 (95% CI 106.70–118.07) per 1,000,000 newborns in China, with a notably higher occurrence in northern China (184.40 per 1,000,000, 95% CI 171.74–197.06) than in southern China (76.77 per 1,000,000, 95% CI 71.02–82.51). Owing to the large sample size and representative regional population distribution, coupled with the lack of publication bias in the literature, our results provide an objective and reliable assessment.

Timely identification, diagnosis, and intervention for OADs via NBS are essential for reducing severe clinical consequences in affected individuals. The widespread application of MS/MS worldwide and improvements in genetic testing technology have enabled prompt OAD detection, diagnosis, and management.

Globally, the incidence of OADs in newborns via NBS varies across regions and is estimated to be 1:16,000 in the United States [62], 1:8000 in the United Kingdom [63], 1:10,000 in Germany [64], 1:3400 in Saudi Arabia [65], and 1:2500 in Iran [66]. In East Asian countries, the estimated incidence rate of OADs in Japan and South Korea is 1:22,000 and 1:31,000, respectively [64]. A nationwide cross-sectional survey of 7 million newborns in mainland China reported an OAD incidence of approximately 1:8000 via NBS [2], consistent with the findings in the present meta-analysis.

Differences in genetic backgrounds between northern and southern China are jointly caused by population migration and environmental factors, as well as genetic drift resulting from geographical isolation [67,68,69]. The genetic differences among the Han Chinese in China show a continuous gradient, following a migration and admixture model from south to north [67]. The hot and humid climate in southern China makes it a high-incidence area for malaria, which has led to a higher gene frequency of G6PD deficiency (favism) as a genetic adaptation to resist malaria [69]. Subgroup analysis in this study demonstrated a significantly higher incidence of OADs in northern China, particularly in the Shandong, Henan, and Hebei provinces, suggesting a geographical trend of higher incidence in northern versus southern China.

Elucidating the local disease spectrum of OADs is important for reproductive counseling, diagnosis, treatment management, integration, and allocation of healthcare resources. Specific disease spectrums of OADs have been identified in different regions and populations. For instance, hydroxymethylglutaric aciduria is predominant in the United Arab Emirates [70], MCCD is prevalent in Austria [71] and Singapore [72], and MMA is prevalent in Japan [64].

In this study, the seven most common diseases—MMA, MCCD, GA-I, IBDD, IVA, PA, and 2-MBD—accounted for 98.07% of all cases. This provides strong evidence for the rapid identification of specific diseases within OADs in the Chinese population. Affected children with MMA and PA suffer from poor feeding, vomiting, and recurrent metabolic decompensation [73]. If not adequately treated, this condition can lead to metabolic acidosis and hyperammonemia, and, in severe cases, may progress to coma or even death. The clinical manifestations of MCCD can vary widely, ranging from asymptomatic individuals to those experiencing acute metabolic crises, including hyperammonemia, hypoglycemia, metabolic acidosis, and neurological abnormalities [74]. Without medical management, most patients with GA1 experience an acute encephalopathic crisis in the first 3–36 months following an intercurrent febrile illness or surgical intervention, resulting in bilateral striatal damage. Patients with IBDD are either asymptomatic, or symptomatic with variable clinical features, including failure to thrive, seizures, anemia, muscular hypotonia, and developmental delay [75]. The clinical manifestations of IVA include paroxysmal vomiting, lethargy or altered mental status, epilepsy, poor feeding, developmental delay, severe metabolic acidosis, hyperammonemia, ketosis, hyper- or hypoglycemia, and cytopenia [76]. Symptomatic patients of 2-MBD present with developmental abnormalities, intellectual disturbance, seizures, muscular atrophy, and even failure to thrive [77].

Furthermore, subgroup analysis revealed a notably higher incidence of MMA and 2-MBD in northern and southern China, respectively, highlighting regional disparities. Additionally, the proportion of patients with MMA combined with homocystinuria was significantly higher in the northern region than in the southern region. Finally, the highest incidence of the disease varied across geographical regions, with 2-MBD predominant in Hunan and Fujian provinces and MMA most prevalent in Shandong, Hebei, Henan, and Gansu Provinces.

This study also has several limitations. First, although our analysis includes studies from 22 provincial-level regions (autonomous regions or municipalities), underreporting persists in regions outside these areas. In addition, the number of newborns included in the studies was also quite limited within some provinces, such as Xinjiang, Beijing, and Shanxi. Second, heterogeneity between studies might reduce the precision of our pooled effect size estimates; thus, caution is required when interpreting our results. Third, our study included only published studies. The exclusion of preprints, conference abstracts, and non-peer-reviewed local or government reports may have introduced publication bias. Finally, all cases of organic acidemias included in the studies demonstrated abnormal results on MS/MS-based NBS and were further confirmed by genetic testing. However, cut-off thresholds for the biochemical markers used in MS/MS-based NBS may vary across laboratories, potentially leading to inconsistent detection of mild cases.

## 5. Conclusions

Our results yielded a comparatively accurate incidence of 112.38 (95% CI 106.70–118.07) per 1,000,000 newborns, highlighting a significantly higher incidence of OADs and MMA in northern China, and a significantly higher incidence of 2-MBD in southern China. Additionally, we confirmed that the ratio of MMA combined with homocystinuria to MMA was higher in northern China than in southern China. Our findings provide valuable epidemiological insights into OADs in the Chinese population, guiding future NBS endeavors for OADs.

## Figures and Tables

**Table 1:** Characteristics of studies included in the meta-analysis.

South or North of China	Reference (Author/ Year)	Area		Years	Screening Cases	OADs Cases	Prevalence	Disease Spectrum of OADs										AHRQ Scores
		**Province**	**City**					MMA	Isolated MMA	MMA Combined with Homocystinuria	PA	GA-I	IVA	MCCD	2-MBD	IBBD	**Other Disease**	
South	Lan et al., 2022 [7]	Fujian	Longyan	2018–2020	58,934	5	1/11,786	N/A	N/A	N/A	1	1	N/A	1	1	1	N/A	9
	Huang et al., 2021 [8]	Fujian	Nanping	2016–2019	74,673	10	1/7467	3	2	1	N/A	1	2	2	1	1	N/A	8
	Zheng et al., 2022 [9]	Fujian	Ningde	2016–2020	135,148	12	1/11,262	N/A	N/A	N/A	N/A	4	1	1	5	1	N/A	8
	Lin et al., 2019 [10]	Fujian	Quanzhou	2014–2018	364,545	39	1/9347	3	N/A	N/A	2	7	4	5	12	6	N/A	9
	Tang et al., 2021 [11]	Guangdong	Guangzhou	2015–2010	272,117	22	1/12,369	10	6	4	1	3	N/A	2	2	3	1	8
	Zhang et al., 2020 [12]	Guangdong	Huizhou	2017–2019	34,689	4	1/8672	2	N/A	N/A	N/A	2	N/A	N/A	N/A	N/A	N/A	7
	Liu et al., 2021 [13]	Guangdong	Meizhou	2019–2021	47,145	3	1/15,715	1	N/A	1	1	N/A	N/A	N/A	N/A	1	N/A	7
	Huang et al., 2024 [14]	Guangdong	Zhongshan	2014–2023	221,731	7	1/31,675	6	4	2	N/A	N/A	1	N/A	N/A	N/A	N/A	8
	Tan et al., 2021 [15]	Guangxi	Liuzhou	2012–2020	111,986	12	1/9332	2	N/A	N/A	2	3	4	N/A	N/A	N/A	1	8
	Tan et al., 2023 [16]	Guangxi	Nanning	2019–2021	16,207	2	1/8103	1	1	N/A	N/A	1	N/A	N/A	N/A	N/A	N/A	7
	Yang et al., 2022 [3]	Guizhou	Guiyang	2015–2019	105,742	2	1/52,871	N/A	N/A	N/A	N/A	N/A	N/A	N/A	N/A	2	N/A	7
	Xie et al., 2020 [17]	Hainan	Haikou	2014–2019	100,158	8	1/12,519	4	N/A	N/A	N/A	N/A	N/A	N/A	N/A	1	3	7
	Xiao et al., 2024 [18]	Hunan	Huaihua	2015–2021	206,977	36	1/5749	2	N/A	N/A	N/A	1	1	1	29	1	1	9
	Li et al., 2022 [19]	Hunan	Changsha	2016–2020	300,849	12	1/25,070	N/A	N/A	N/A	3	1	2	3	3	N/A	N/A	8
	Luo et al., 2024 [20]	Hunan	Zhuzhou	2019–2022	90,829	3	1/30,276	1	N/A	N/A	N/A	1	N/A	N/A	N/A	N/A	1	8
	Liu et al., 2020 [21]	Jiangsu	Changzhou	2019–2022	50,844	7	1/7263	2	N/A	N/A	N/A	3	N/A	2	N/A	N/A	N/A	7
	Huang et al., 2024 [22]	Jiangsu	Jiangyin	2019–2022	41,058	5	1/8211	2	N/A	2	1	N/A	N/A	N/A	1	1	N/A	7
	Yang et al., 2019 [23]	Jiangsu	Nanjing and Yangzhou	2014–2028	536,008	42	1/12,762	15	N/A	N/A	1	6	3	15	1	N/A	1	9
	Zhang et al., 2022 [24]	Jiangsu	Suqian	2019–2022	204,604	20	1/10,230	9	3	6	1	1	N/A	6	N/A	3	N/A	8
	Wang et al., 2019 [25]	Jiangsu	Suzhou	2014–2018	401,660	33	1/12,171	10	7	3	1	4	2	12	N/A	3	1	9
	Wang et al., 2022 [26]	Jiangxi	Nanchang	2016–2021	126,111	7	1/18,105	3	N/A	N/A	N/A	N/A	1	2	N/A	1	N/A	7
	Yang et al., 2021 [27]	Jiangxi	Shangrao	2016–2021	15,207	1	1/15,207	N/A	N/A	N/A	N/A	N/A	N/A	1	N/A	N/A	N/A	7
	Zhang et al., 2020 [28]	Sichuan	Chengdou	2017–2018	39,648	6	1/6608	2	N/A	N/A	1	N/A	1	1	1	N/A	N/A	7
	Shi et al., 2024 [29]	Sichuan	Yibin	2019–2021	71,572	5	1/14,314	N/A	N/A	N/A	N/A	N/A	2	N/A	N/A	1	2	7
	Yu et al., 2024 [30]	Zhejiang	Ningbo	2014–2023	555,129	36	1/15,402	13	N/A	N/A	1	N/A	1	18	N/A	3	N/A	9
	Zhao et al., 2021 [31]	Zhejiang	Except Ningbo	2009–2018	3,040,815	208	1/14,619	78	N/A	N/A	11	16	18	48	5	29	3	9
	Hao et al., 2024 [32]	Shanghai		2003–2022	1,176,073	115	1/10,226	56	21	35	8	3	8	28	1	10	1	9
	Zhang et al., 2024 [33]	Shanghai		2010–2020	232,561	12	1/19,308	5	N/A	N/A	1	2	3	1	N/A	N/A	N/A	8
	Lee et al., 2011 [34]	Hongkong		2005–2009	177,246	5	1/35,449	1	N/A	N/A	N/A	1	1	N/A	N/A	N/A	2	7
	Belaramani et al., 2024 [35]	Hongkong		2015–2022	125,688	7	1/17,955	2	N/A	N/A	2	N/A	1	N/A	N/A	N/A	2	6
North	Men et al., 2023 [36]	Jiangsu	Lianyungang	2015–2021	245,194	22	1/11,145	9	N/A	N/A	3	N/A	1	5	1	2	1	8
	Zhou et al., 2018 [37]	Jiangsu	Xuzhou	2015–2017	165,262	13	1/12,712	12	11	1	N/A	1	N/A	N/A	N/A	N/A	N/A	7
	Wang et al., 2018 [38]	Gansu		2015–2016	77,957	4	1/19,489	3	N/A	N/A	N/A	N/A	1	N/A	N/A	N/A	N/A	7
	Liu et al., 2024 [39]	Gansu		2021–2023	213,786	28	1/7635	24	10	14	1	N/A	N/A	2	N/A	N/A	1	8
	Zhang et al., 2024 [40]	Gansu		2017–2020	286,682	41	1/6992	32	15	17	1	1	N/A	5	N/A	N/A	2	9
	Feng et al., 2020 [41]	Hebei	Shijiazhuang	2014–2018	128,399	36	1/3566	34	3	31	N/A	N/A	N/A	2	N/A	N/A	N/A	9
	Ma et al., 2020 [42]	Henan		2013–2019	850,486	200	1/4252	182	21	161	3	4	1	10	N/A	N/A	N/A	9
	Ma et al., 2020 [43]	Henan	Xinxiang	2015–2018	50,112	9	1/5568	8	N/A	N/A	N/A	N/A	1	N/A	N/A	N/A	N/A	7
	Zhu et al., 2020 [44]	Jilin	Changchun	2015–2018	105,437	5	1/21,087	3	N/A	N/A	N/A	2	N/A	N/A	N/A	N/A	N/A	7
	Liu et al., 2017 [45]	Jilin	Liaoyuan	2011–2016	10,503	1	1/10,503	1	N/A	N/A	N/A	N/A	N/A	N/A	N/A	N/A	N/A	6
	Mao et al., 2020 [46]	Xingxia		2016–2019	189,354	15	1/12,623	7	N/A	N/A	1	N/A	2	2	N/A	N/A	3	7
	Wang. 2021 [4]	Shandong	Heze	2015–2020	266,609	120	1/2221	106	6	100	N/A	1	1	12	N/A	N/A	N/A	9
	Duan et al., 2024 [47]	Shandong	Jining	2022	52,192	8	1/6524	4	1	3	1	N/A	N/A	3	N/A	N/A	N/A	7
	Yang et al., 2020 [48]	Shandong	Jining	2014–2018	514,234	126	1/4081	91	23	68	6	3	2	20	N/A	4	N/A	9
	Zhao et al., 2022 [49]	Shandong	Langfang	2020	41,062	4	1/10,265	2	N/A	N/A	N/A	1	N/A	1	N/A	N/A	N/A	7
	Zhang et al., 2024 [50]	Shandong	Liaocheng	2020–2021	22,457	4	1/5614	2	N/A	N/A	N/A	N/A	N/A	2	N/A	N/A	N/A	6
	Xia, 2018 [51]	Shandong	Linyi	2014–2017	83,570	6	1/13,928	5	2	3	N/A	1	N/A	N/A	N/A	N/A	N/A	7
	Yin, 2022 [52]	Shandong	Taian	2013–2019	222,754	23	1/9684	13	N/A	N/A	N/A	4	2	4	N/A	N/A	N/A	8
	Ju et al., 2020 [53]	Shandong	Weihai	2018–2019	44,438	5	1/8887	3	N/A	3	N/A	N/A	N/A	1	1	N/A	N/A	7
	Pei et al., 2018 [54]	Shandong	Weifang	2013–2016	77,974	14	1/5570	12	N/A	N/A	N/A	1	N/A	1	N/A	N/A	N/A	8
	Zhao et al., 2024 [55]	Shandong	Zaozhuang	2015–2023	183,741	19	1/9670	16	2	14	N/A	N/A	1	2	N/A	N/A	N/A	9
	Mou et al., 2023 [56]	Shandong	Zibo	2015–2022	223,368	51	1/4379	45	3	42	1	1	2	1	1	N/A	N/A	9
	Liu et al., 2023 [57]	Shandong	Rizhao	2016–2022	36,590	8	1/4573	3	2	1	4	N/A	N/A	N/A	N/A	1	N/A	7
	Zhang et al., 2019 [58]	Shanxi	Taiyuan	2016–2022	81,138	7	1/11,591	3	N/A	N/A	N/A	N/A	N/A	N/A	N/A	2	2	7
	Zhang et al., 2021 [59]	Shanxi	Xian	2014–2019	146,152	28	1/5219	21	11	10	N/A	2	N/A	3	N/A	1	1	9
	Zhu et al., 2024 [60]	Xinjiang	Xulumuqi	2018–2021	41,690	9	1/4632	5	3	2	1	1	1	1	N/A	N/A	N/A	7
	Yang et al., 2020 [61]	Beijing		2014–2019	58,651	9	1/6516	7	2	5	1	N/A	1	N/A	N/A	N/A	N/A	7

OAD, organic acid disorder; N/A, not available.

## Data Availability

All data generated or analyzed during this study are included in the article; further inquiries can be directed to the corresponding author.

## Supplementary Materials

The following supporting information can be downloaded at: https://www.mdpi.com/article/10.3390/ijns11040113/s1: Figure S1: Meta-analysis of the prevalence of GA-I between southern and northern China; Figure S2: Meta-analysis of the prevalence of IBBD between southern and northern China; Figure S3: Meta-analysis of the prevalence of IVA between southern and northern China; Figure S4: Meta-analysis of the prevalence of MCCD between southern and northern China; Figure S5: Meta-analysis of the prevalence of P between southern and northern China. Table S1: The Agency for Healthcare Research and Quality (AHRQ) methodology checklist.

## Abbreviations

The following abbreviations are used in this manuscript:

OADs	Organic acid disorders
NBS	Newborn screening
MS/MS	Tandem mass spectrometry
MMA	Methylmalonic acidemia
MCCD	3-methylcrotonyl-CoA carboxylase deficiency
GA-I	Glutaric acidemia type I
IBDD	Isobutyryl-CoA dehydrogenase deficiency
IVA	Isovaleric acidemia
2-MBD	2-methylbutyryl-CoA dehydrogenase deficiency
PA	Propionic acidemia
HCS	Holocarboxylase synthetase deficiency
BTD	Biotinidase deficiency
