# Supplementary material for: Incidence of Organic Acid Disorders in 13 Million Chinese Newborns: A Systematic Review and Meta-Analysis
Source: Int J Neonatal Screen. 2025 Dec 13;11(4):113. doi: 10.3390/ijns11040113 (PMC12734155; doi:10.3390/ijns11040113)
Supplement: Supplementary file 1 [file IJNS-11-00113-s001.zip › IJNS-3906226-Table S1.pdf]

Table S1 The Agency for Healthcare Research and Quality (AHRQ) methodology checklist

#	Item	Question	Yes	No	Unclear
1	Source of Information	Was the source of data (e.g., patients, medical records) clearly described and appropriate?			
2	Inclusion/Exclusion Criteria	Were the inclusion and exclusion criteria pre-specified and clearly stated?			
3	Time Frame	Was the period of patient recruitment or data collection specified?			
4	Representativeness	Was the study population representative of a defined community or source population?			
5	Sample Size Justification	Was there an explanation of how the sample size was determined (e.g., power calculation)?			
6	Assessment of Exposure	Was the exposure assessed in a valid and reliable way for all participants?			
7	Temporal Relationship	If comparing groups, was it clear that the outcome was not present at baseline?			
8	Different Levels of Exposure	If relevant, were different levels (doses) of the exposure assessed?			
9	Confounding	Were potential confounders identified and strategies to address them described (e.g., multivariate analysis)?			
10	Assessment of Outcome	Was the outcome assessed in a valid and reliable way for all participants?			
11	Follow-up & Attrition	Was follow-up time sufficient and loss-to-follow-up reported and accounted for?			
