# Supplementary material for: Incidence of Organic Acid Disorders in 13 Million Chinese Newborns: A Systematic Review and Meta-Analysis
Source: Int J Neonatal Screen. 2025 Dec 13;11(4):113. doi: 10.3390/ijns11040113 (PMC12734155; doi:10.3390/ijns11040113)
Supplement: Supplementary file 1 [file IJNS-11-00113-s001.zip › Supplementary Figure Legends.pdf]

Figure Legends

Figure S1 Meta-analysis of the prevalence of GA-I between southern and northern China.

Figure S2 Meta-analysis of the prevalence of IBBD between southern and northern China.

Figure S3 Meta-analysis of the prevalence of IVA between southern and northern China.

Figure S4 Meta-analysis of the prevalence of MCCD between southern and northern China.

Figure S5 Meta-analysis of the prevalence of P between southern and northern China.
