# Supplementary figures and images for: Incidence of Organic Acid Disorders in 13 Million Chinese Newborns: A Systematic Review and Meta-Analysis
Source: Int J Neonatal Screen. 2025 Dec 13;11(4):113. doi: 10.3390/ijns11040113 (PMC12734155; doi:10.3390/ijns11040113)

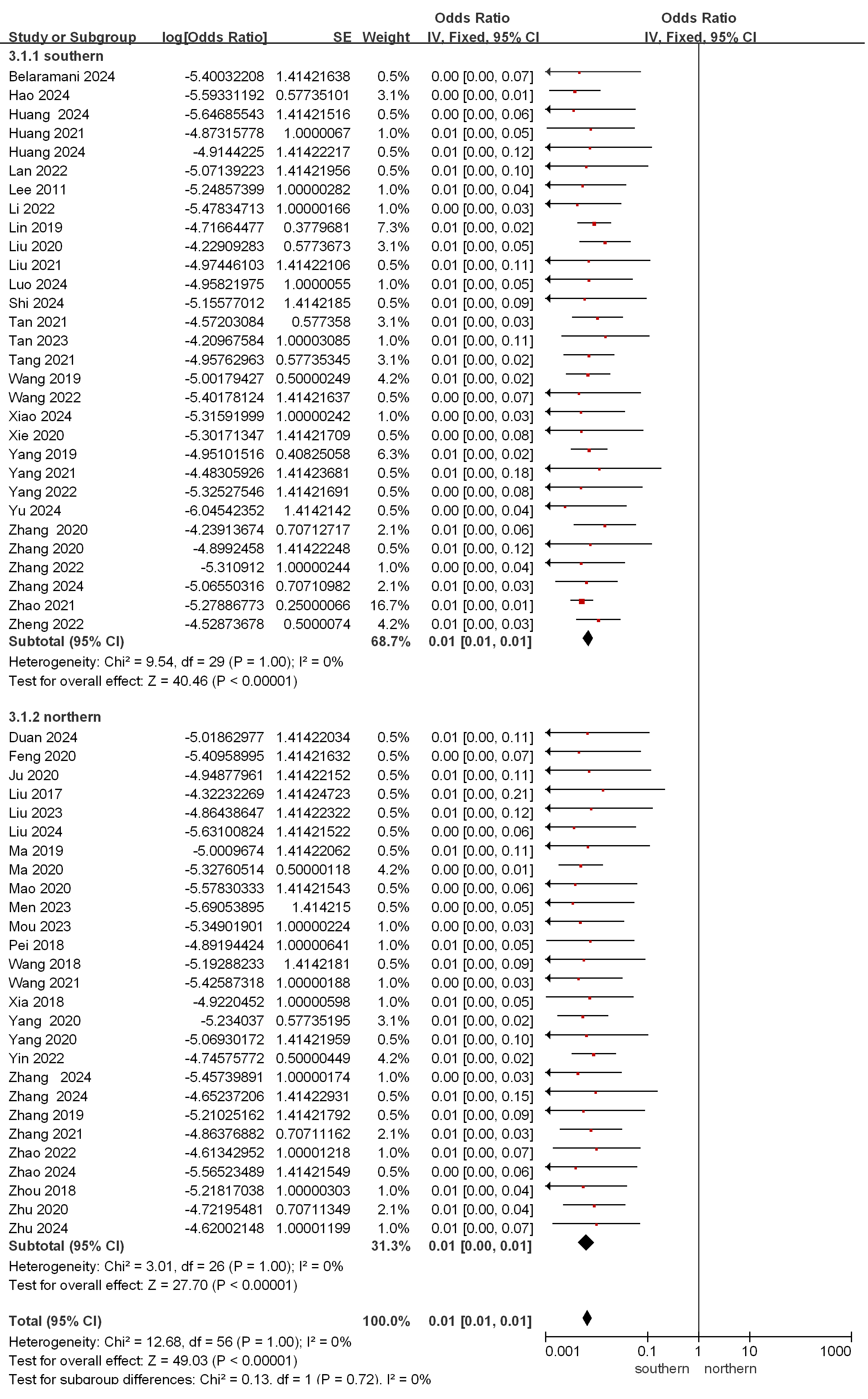

Supplement: Supplementary file 1 [file IJNS-11-00113-s001.zip › Figure S1.tif]

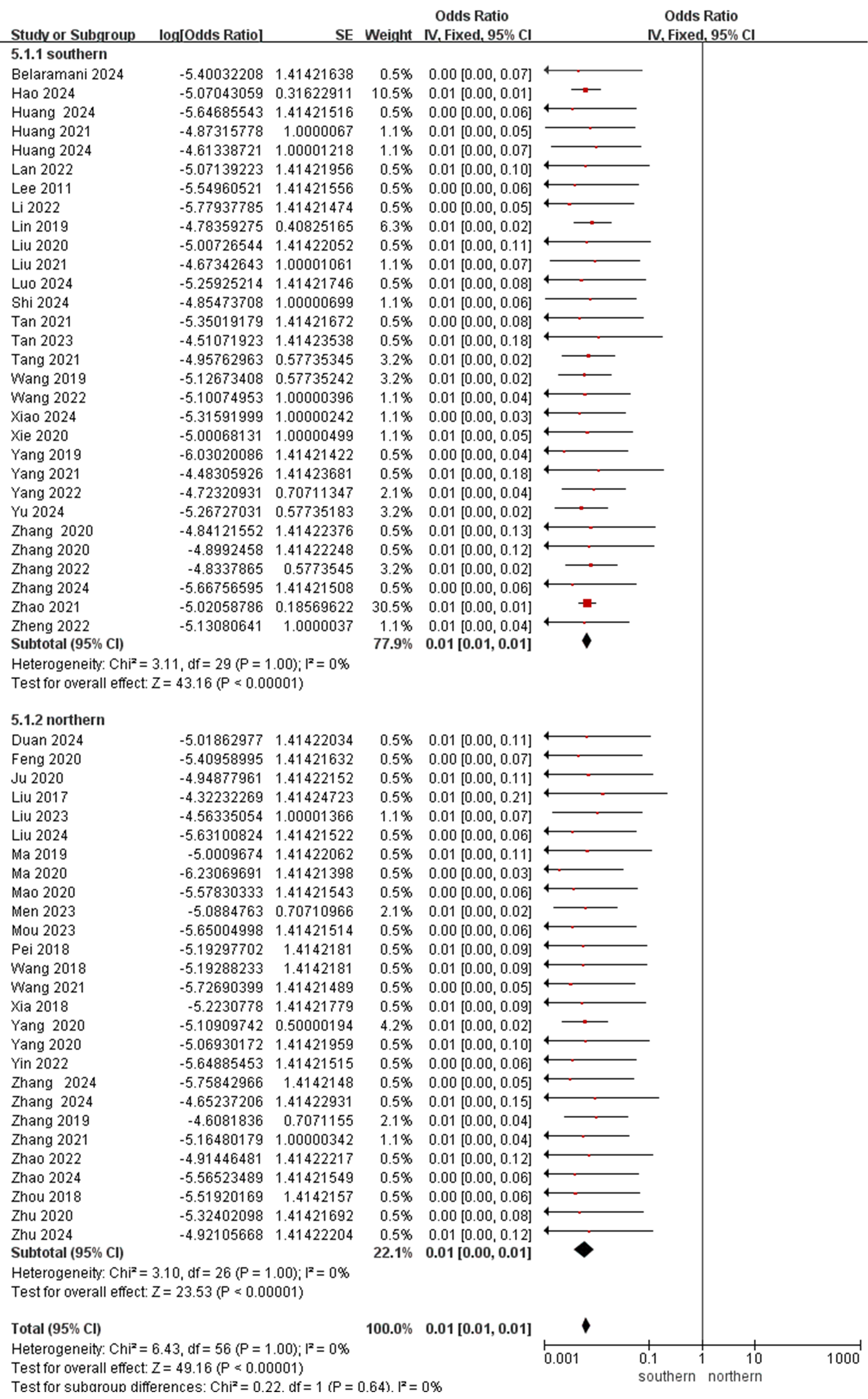

Supplement: Supplementary file 1 [file IJNS-11-00113-s001.zip › Figure S2.tif]

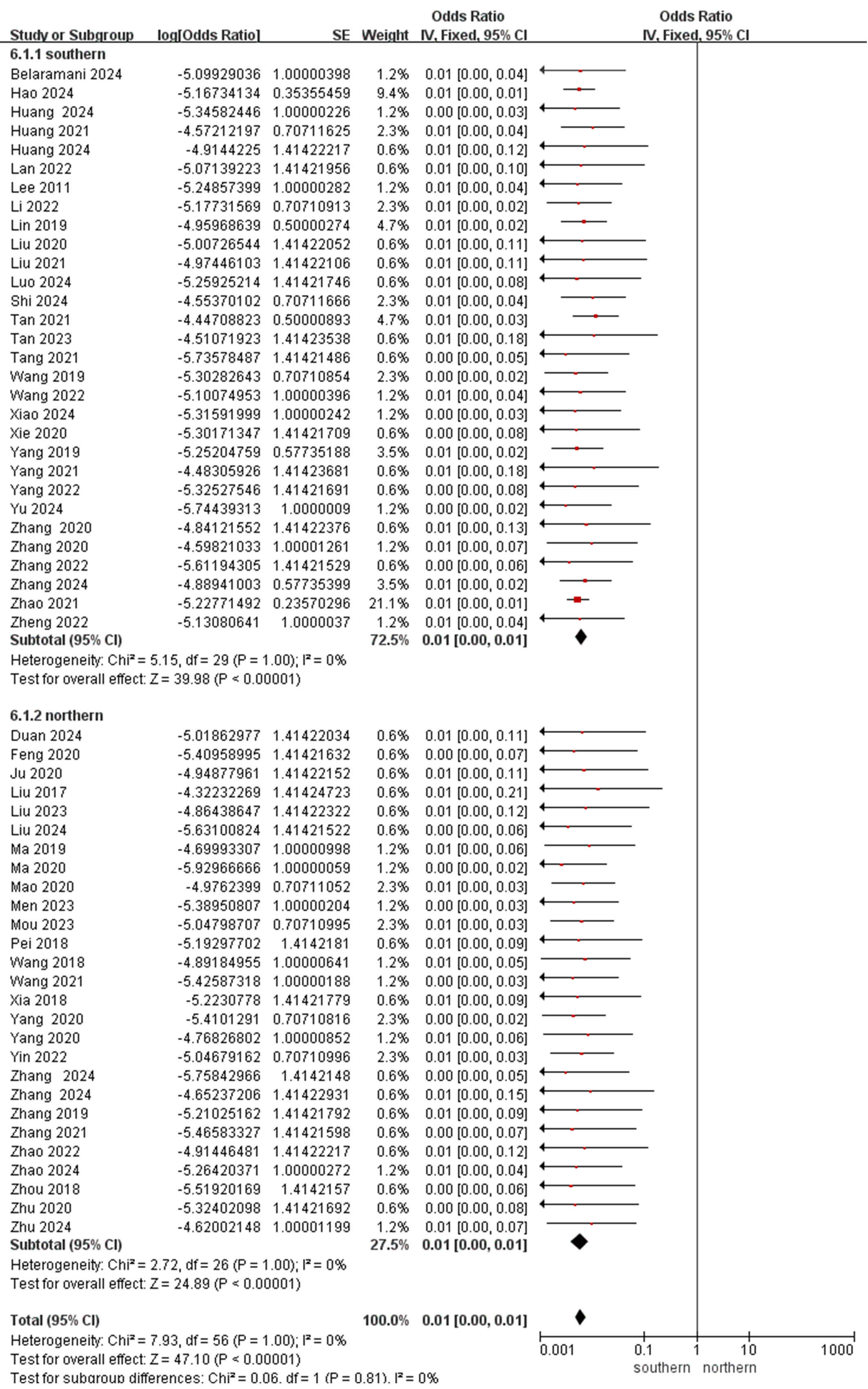

Supplement: Supplementary file 1 [file IJNS-11-00113-s001.zip › Figure S3.tif]

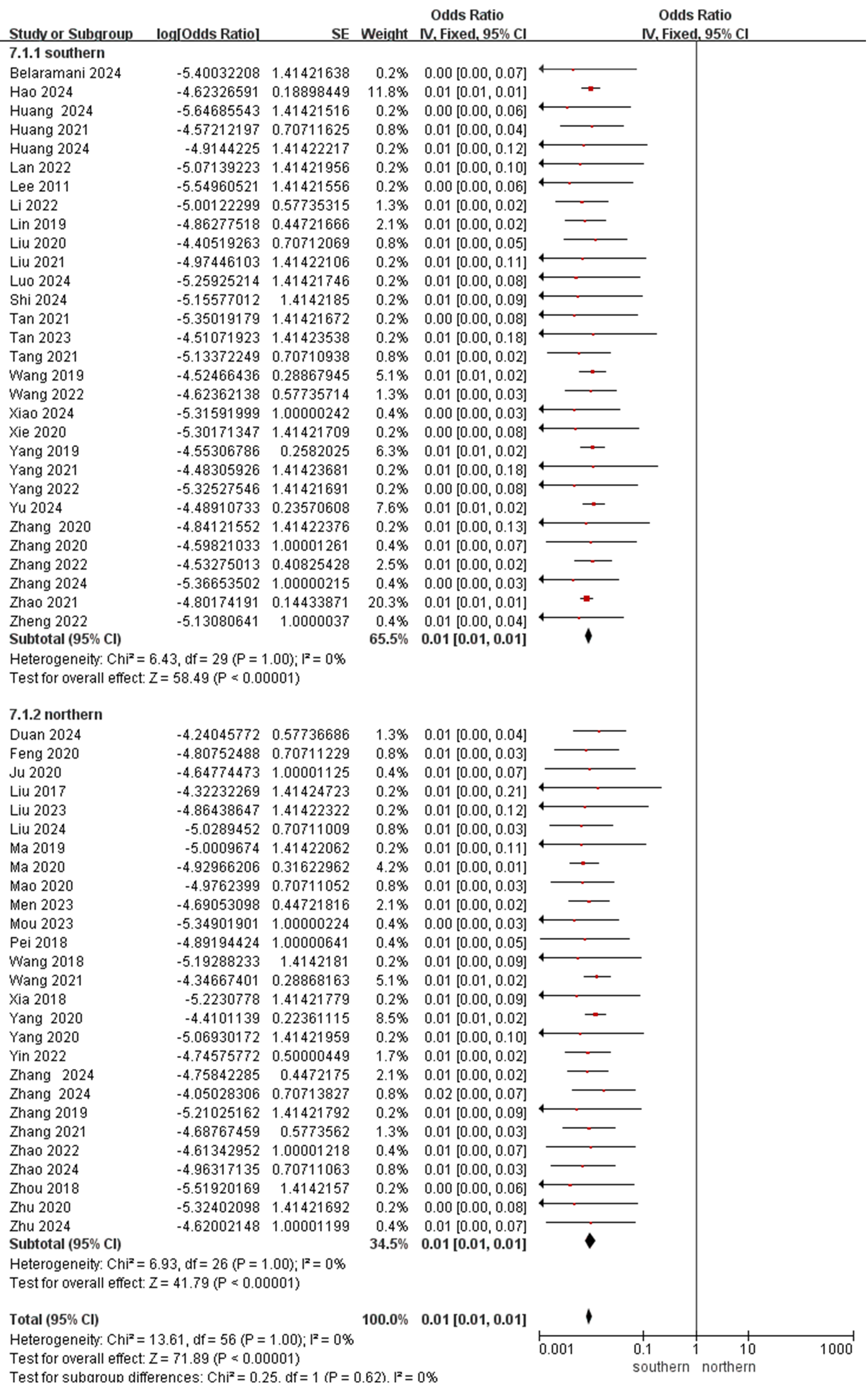

Supplement: Supplementary file 1 [file IJNS-11-00113-s001.zip › Figure S4.tif]

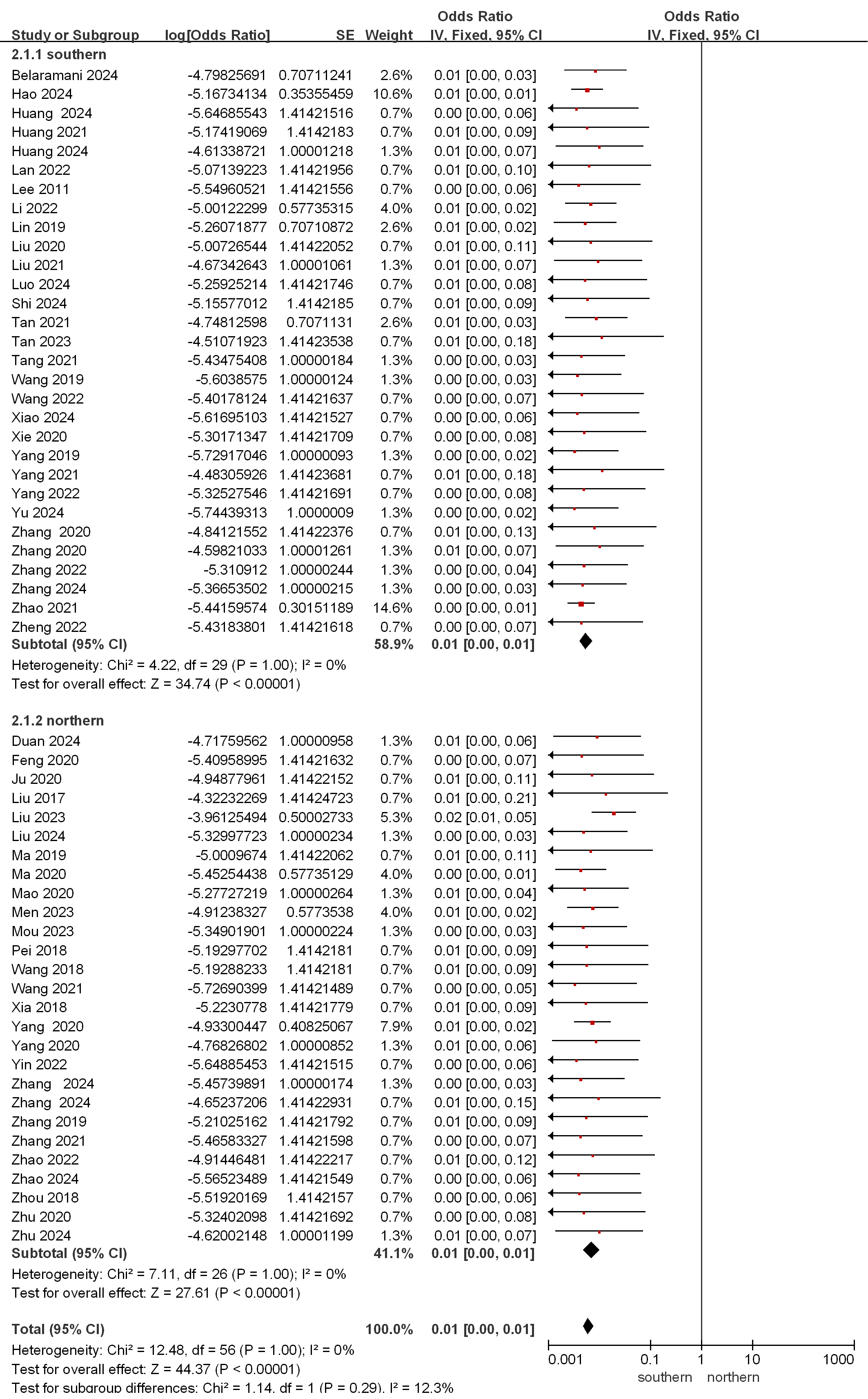

Supplement: Supplementary file 1 [file IJNS-11-00113-s001.zip › Figure S5.tif]
